# Use of an Internet-of-Things Smart Home System for Healthy Aging in Older Adults in Residential Settings: Pilot Feasibility Study

**DOI:** 10.2196/21964

**Published:** 2020-11-10

**Authors:** Yong K Choi, Hilaire J Thompson, George Demiris

**Affiliations:** 1 Department of Public Health Sciences School of Medicine University of California Davis Sacramento, CA United States; 2 School of Nursing University of Washington Seattle, WA United States; 3 Biobehavioral Health Sciences School of Nursing University of Pennsylvania Pennsylvania, PA United States

**Keywords:** Internet of Things, smart home, independent living, aging, healthy aging

## Abstract

**Background:**

The Internet-of-Things (IoT) technologies can create *smart* residences that integrate technology within the home to enhance residents’ safety as well as monitor their health and wellness. However, there has been little research on real-world testing of IoT smart home devices with older adults, and the feasibility and acceptance of such tools have not been systematically examined.

**Objective:**

This study aims to conduct a pilot study to investigate the feasibility of using IoT smart home devices in the actual residences of older adults to facilitate healthy aging.

**Methods:**

We conducted a 2-month feasibility study on community-dwelling older adults. Participants chose among different IoT devices to be installed and deployed within their homes. The IoT devices tested varied depending on the participant’s preference: a door and window sensor, a multipurpose sensor (motion, temperature, luminosity, and humidity), a voice-operated smart speaker, and an internet protocol (IP) video camera.

**Results:**

We recruited a total of 37 older adults for this study, with 35 (95%) successfully completing all procedures in the 2-month study. The average age of the sample was 78 (SD 9) years and primarily comprised women (29/37, 78%), those who were educated (31/37, 86%; bachelor’s degree or higher), and those affected by chronic conditions (33/37, 89%). The most widely chosen devices among the participants were multipurpose sensors and smart speakers. An IP camera was a significantly unpopular choice among participants in both phases. The participant feedback suggests that perceived privacy concerns, perceived usefulness, and curiosity to technology were strong factors when considering which device to have installed in their home.

**Conclusions:**

Overall, our deployment results revealed that the use of IoT smart home devices is feasible in actual residences of older adults. These findings may inform the follow-up assessment of IoT technologies and their impact on health-related outcomes and advance our understanding of the role of IoT home-based monitoring technologies to promote successful aging-in-place for older adults. Future trials should consider older adults’ preferences for the different types of smart home devices to be installed in real-world residential settings.

## Introduction

### Background

*Aging-in-place* is a concept that has been proposed to address older adults’ needs and expectations of successful aging. It calls for supporting older adults’ desire to remain and live in their own homes without having to relocate to support facilities such as nursing homes [1]. The importance of aging-in-place has been shown consistently across different surveys of older adults [1]. However, while older adults show a strong desire to stay independent without having to give up their own lifestyle as they age, aging-in-place can introduce some challenges. These include managing one’s own health, performing various activities of daily living, and maintaining social connections while experiencing health-related changes. Previous research has shown that older adults who live alone can face issues related to isolation, mobility, hygiene, finances, health management, home management, safety, and nutrition [2-4]. Consequently, there is an increased need for interventions to support the successful aging-in-place of older adults. To this end, there has been a growing interest in the use of technologies for older adults, including those that can facilitate health monitoring of older adults in their residence to promote healthy aging in their own homes. The need for this type of technological intervention is amplified by the growing older adult population, increasing health care needs, and the desire of older adults to age in their own homes.

The Internet of Things (IoT) is a network of objects (eg, sensors, appliances, cars) equipped with internet connectivity, enabling them to send and receive data [5]. Therefore, IoT objects or devices can interact with each other and cooperate to provide value-added services to the user [6]. One of the most prominent examples of IoT is the smart home. An IoT smart home can consist of smart appliances (eg, washers, dryers, refrigerators), smart home safety and security systems (sensors, monitors, cameras, and alarm systems), and smart home energy equipment, such as smart thermostats and smart lighting. Such IoT devices can create *smart* residences that integrate technology within the home to enhance residents’ safety as well as monitor their health and wellness. The residences equipped with IoT smart home devices potentially make the lives of older adults easier, more convenient, and safer. For example, older adults with limited mobility will be able to control their doors, window blinds, or light switches by simply giving voice commands. For these older adults, being empowered to perform these daily activities on their own is the difference between being able to live independently or needing assistance at home or moving to an assisted living facility. In addition, the advancement in IoT sensor technologies along with advanced data analytics presents an opportunity to support independent aging by identifying potential patterns in health, detecting anomalous activities, and prompting early intervention to prevent adverse health events [7-11].

The use of home-based sensor technologies to passively monitor activity levels of older adults is a concept that has been tested previously [10,12-22]. Previous research has shown that such technologies could accurately detect abnormal movement or behaviors [10,21-24], and older adults are interested in receiving data from sensor technologies that provide better insight into their health status [25,26]. In addition, older adults have demonstrated their belief that sensor-based passive monitoring systems in their homes have the potential to enhance their quality of life [27]. Although these projects provide initial insights into the potential of health monitoring using smart home sensors, most of these efforts were not real-world evaluation studies with older adults and did not assess older adults’ preferences for diverse arrays of IoT smart home devices. In addition, previous research has used systems with hardware components that capture and transmit data but do not have ways to interact with other devices and aggregate the data in a central repository, as would be the case in an IoT-based smart home system. To our knowledge, there has been little research on real-world testing of IoT smart home devices with older adults, and the feasibility and acceptance of such tools have not been systematically examined. Therefore, the purpose of this study is to address this gap by conducting a pilot study to investigate the feasibility of using IoT-based smart home devices in actual residences of older adults.

### Objectives

Our objective is to investigate the feasibility of using IoT smart home devices in real-world residential settings of older adults. To demonstrate feasibility, we assessed the following key aspects of future trial design: (1) recruitment and retention, (2) participants’ preference for device choices, (3) device deployment and maintenance, (4) feasibility of data collection, and (5) acceptability of the selected health outcome measures. As this was a feasibility study, no controls or randomization was used, and no specific interventions were administered during the study. All study procedures were approved by the University of Washington, Institutional Review Board.

## Methods

### Study Design

This study was a 2-month feasibility study that enrolled community-dwelling older adults in the Puget Sound area to choose among different IoT devices to be installed and deployed within their homes. The devices varied depending on the preference of the participant, and options included the following: (1) a door and window sensor, (2) a multipurpose sensor (motion, temperature, and luminosity), (3) a voice-operated smart speaker, and (4) an IP video camera (see *IoT Device Description and Deployment* for more detail). Over the study period, participants were interviewed at 3 different time points: baseline, 1 month, and 2 months (study exit) to understand their thoughts about the devices.

We recruited participants through collaboration with local retirement communities in the Puget Sound area. In order to be eligible for the study, participants needed to be (1) community-dwelling older adults, (2) able to read and write English, (3) have an internet connection at their residence, and (4) choose at least one or more devices for installation in the home. Recruitment occurred at 6 different senior housing communities to include individuals across a range of lower to middle-upper socioeconomic status. The communities house older adults who have the capacity to live independently with minimal help in maintaining their home or activities of daily living. Working with facility administrators, we posted recruitment flyers and held information sessions that included a short presentation about the research project, followed by a question and answer session. After the presentation, interested individuals either went through an informed consent process with study team members or filled out contact information to be contacted later for enrollment in the study. In the latter case, informed consent was obtained during the baseline visit before any study procedures. We also conducted snowball sampling to identify potential participants who may be interested in participation. During the informed consent process, the subject chose the devices to be installed and indicated their choice on the form. To compensate participants for their time, we provided US $25 gift cards following the first- and second-month interview visits.

In this study, recruitment occurred in 2 different phases. For phase 1, a voice-operated smart speaker was not one of the available IoT devices, and eligible participants had to be living alone on top of the aforementioned inclusion criteria. For phase 2, we added the option of a voice-operated smart speaker and made it eligible for interested couples who live together to join the study together. The recruitment process and the study procedures remained the same between the 2 phases. In total, 37 participants were included in the study. Fifteen participants were recruited during phase 1 (12 females and 3 males) and 22 participants were recruited during phase 2 (17 females and 5 males).

### IoT Device Description and Deployment

**Figure 1:**
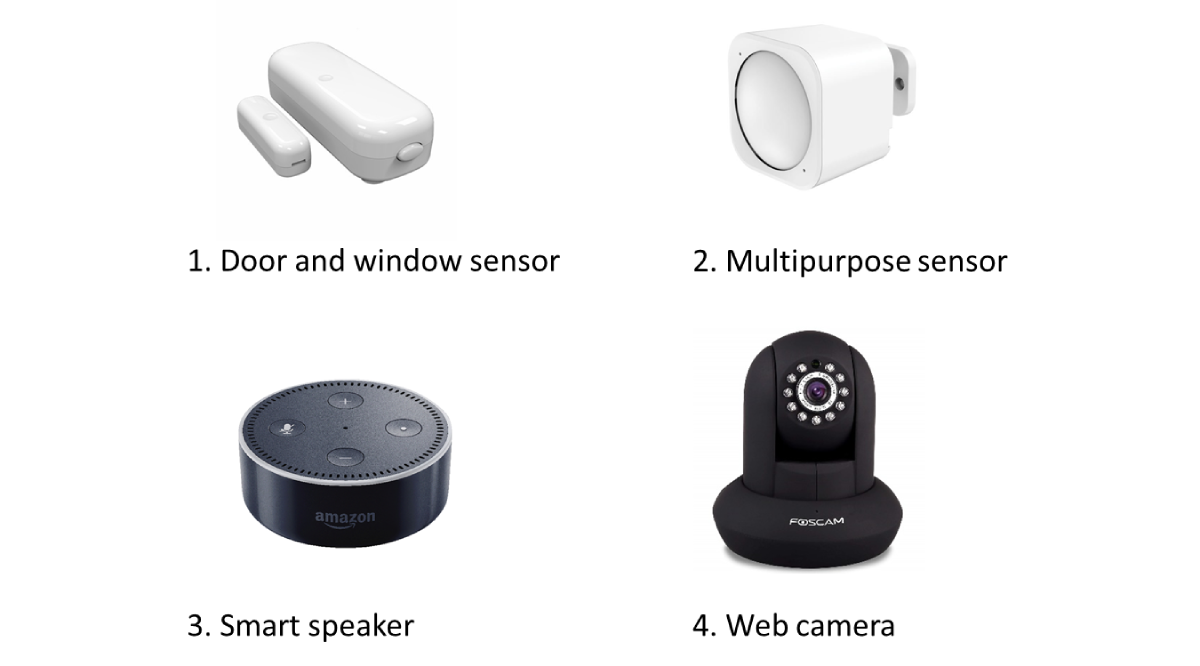
Device pictures.

Table 1 provides an overview of the IoT devices available for the participants to choose and evaluate for this study. Figure 1 presents pictures of the devices used in the study. All the devices were commercially available. The primary investigator conducted installations of the devices and provided technical support via phone or by making in-home visits during the duration of the study when necessary. The frequency and reasons for additional visits outside the scheduled study visits were recorded. Participants were also encouraged to contact the primary investigator if they had any questions or issues related to the devices.

**Table 1:** Internet-of-Things smart home devices used in this study.

Device	Data collected	Data transfer protocol	Location of deployment within the home
Door and window sensor	Binary on or off signal when the switch is activated	Z-wave	Front door, refrigerator
Multipurpose sensor	Luminosity, temperature, humidity, and motion	Z-wave	Living room, bedroom
Voice-operated smart speaker	The transcripts of the questions and requests made during the study period	Wi-Fi	Living room
Internet protocol web camera	Live video streaming. No video recording was collected	Wi-Fi	Living room, bedroom

#### Door and Window Sensor and Multipurpose Sensor Deployment

Door and window sensor and multipurpose sensor: The door and window sensor record a binary on or off signal when the magnetic switch is activated. The multipurpose sensor collects data on motion, temperature, luminosity, and humidity. The door and window sensor were installed either at the front entrance of the residence or the fridge. The multipurpose sensor was installed in the living room area or the bedroom depending on the preference of the participant. To deploy these 2 sensors, we used the open source software platform called *Lab of Things* developed by Microsoft Research [28,29]. This platform was installed in a small study laptop and deployed together with a door and window sensor and a multipurpose sensor. For communication between the Lab of Things platform and the sensors, a Z-wave USB dongle was attached to the laptop. During the study, the laptop was plugged into the outlet and left on all the time for processing and sending the sensor data to our research cloud server. The laptop was closed lid and placed to be as unobtrusive as possible to the participant’s home.

#### Voice-Controlled Smart Speaker Deployment

The smart speakers are equipped with a far-field microphone that supports voice recognition. This allows for various hands-free operations, including playing music, retrieving information, and setting reminders and alarms. For this study, we used Echo Dot, a smart speaker manufactured by Amazon. Amazon smart speakers provide capabilities or skills that enable users to try out features created by third-party designers and developers for a more personalized experience. For example, *WebMD*’s skill allows users to ask basic health-related questions. The initial training of how to use the smart speaker was provided by a member of the research team. The participants were encouraged to explore various features during the study period and consider the potential uses of a smart speaker in the management of health context. In addition to the initial training, a list of basic commands was provided to the participants to facilitate the usage of the smart speaker.

#### IP Web Camera Deployment

The use of an internet protocol (IP) camera allows for the synchronous monitoring of a room or other area in the home by the participants. The camera was installed in the living room area or the bedroom location according to the preference of the participants. The participants had the option to have the accompanying monitoring app installed on their mobile phone or just use a regular desktop browser to view the live feed from the camera. The research team did not monitor the live feed from the camera because providing a monitoring service was not the goal of this study. However, the participants could choose to share access to the camera with someone in their life by sharing the web address of the secured camera dashboard and the accompanying ID and password.

### Procedures

#### Baseline Visit

Once participants agreed to participate in the study and provided written informed consent, we scheduled an in-person appointment with the participant for the baseline visit. During the baseline visit, we installed participant-selected IoT devices in the subject’s residence. Installation took approximately 30-45 mins if a participant was to select all offered devices. After the installation was complete, we collected demographic data including age, gender, marital status, education, insurance status, history of chronic conditions and current medications, and the use of assistive devices. In addition, we administered the eHealth literacy scale (eHEALS) [30] to measure one’s comfort level with the technology. Health-related data that incorporate physical, psychosocial, functional, and mobility-related parameters were collected using validated self-report instruments. For a complete description of instruments and the data collection schedule, see the *Data Collection and Analysis* section. After all the questionnaire data were collected, a semistructured interview was conducted to assess the initial participant perspectives on IoT smart home devices. The questionnaires and interview questions took 30-45 mins, and in conjunction with installation, the first visit lasted between 60 and 90 mins in total.

#### Midpoint (1-Month) Visit

During the midpoint visit, we conducted an in-person interview to assess the perceived usefulness of the installed IoT smart home technology, any challenges, privacy or other concerns, and any recommendations or feedback that subjects had at this point. During this visit, we presented participants with graphs of their own sensor data collected during the first month, asking for thoughts and feedback (see the *Smart Home Activity Data Visualization* section below). The visit lasted 30 to 45 mins.

#### Exit (2-Month) Visit

After 2 months, we conducted an exit visit in the subjects’ homes. The installed devices were removed at the beginning of the visit. We administered questionnaires and conducted a semistructured interview to assess perceived obtrusiveness of the IoT smart home technology, any challenges, privacy or other concerns, and any recommendations or feedback (pertaining to their overall experience) subjects may have as they concluded their participation. The exit visit took approximately 60 mins. All interviews at the 3 timepoints were digitally audio-recorded and transcribed using a professional transcription service.

#### Smart Home Activity Data Visualization

Participants who selected motion tracking sensors (eg, a door and window sensor, a multipurpose sensor) were presented with graphs of their own sensor data obtained from the motion sensors. The line graphs and bar graphs were created by PI by aggregating the sensor data using R software to show the activity trends and pattern changes over time. The number of graphs shown to the participants varied based on the selection of devices. Participants who selected a smart speaker or an IP camera did not see the graphs, and no questions were asked related to visualization.

### Data Collection and Analysis

Multimedia Appendix 1 [23-27] outlines the instruments used for this study and the data collection schedule. Instruments were selected to test for feasibility of data collection and acceptability for measuring health status outcomes for future smart home studies. For the analysis of demographics and the selection of IoT devices at baseline, we used descriptive statistics. In addition, we used the paired two-tailed *t* test and chi-square test to compare the pre-post assessments of participants’ health-related variables in an exploratory manner, as the study was not powered to detect statistically significant changes over time. The statistical software program R was used to complete the quantitative data analyses. Interview sessions (at baseline, midpoint, and exit) were audio-recorded and transcribed. Descriptive content analysis [31] of the interview data was performed by at least two independent researchers, and the validity of the interpretations was checked by a third trained member.

## Results

### Recruitment and Retention

A total of 51 people inquired about the study, expressing initial interest to join after attending the recruitment information session or contacting the research team member using the recruitment flyer. Among the 51 inquiries, 47 were from the information session, 2 from the study flyer contact information, and 2 were contacts from snowball sampling from the enrolled participants. Table 2 summarizes the recorded reasons that were identified to exclude participation.

We recruited a total of 37 older adults for this study (15 in phase 1 and 22 in phase 2). For those who were recruited, one participant (ph1_p1) during phase 1 did not complete the full 2-month study, dropping out after completing the midpoint visit. This individual mentioned very low perceived utility of the devices and complained about unidentified technical issues experienced at home. Another participant (ph1_p7) was lost to follow-up for the midpoint visit but contact was re-established for the exit interview. All other participants (n=35) successfully completed all the procedures in the 2-month study.

**Table 2:** Reasons for exclusion.

Reasons for exclusion from the study	Number of people (n=14), n (%)
Does not live alone^a^	2 (14)
Younger than 65 years	4 (29)
No internet connection at home	5 (36)
Lost to follow-up contact or no reasons recorded	3 (21)

^a^Phase 1 required people to live alone to be eligible. This criterion was relaxed in phase 2 recruitment.

### Device Selection by the Participants

Table 3 shows the choice of IoT device selection by the participants. Among the phase 1 group, the most widely chosen device was the multipurpose sensor (14/15, 93%), closely followed by the door and window sensor (12/15, 80%) in phase 1. Among the phase 2 group participants, a smart speaker (19/22, 86%) was the most widely chosen device, followed by the multipurpose sensor (18/22, 81%) and the door and window sensor (15/22, 68%). An IP camera was a significantly unpopular choice among participants in both phases (phase 1: 2/15, 13%; phase 2: 3/22, 13%). Most participants commented that the potential privacy risks deterred them from choosing the camera. For both phases, the participants had options to choose more than one device. The most frequently selected combinations of devices for phase 1 were door and window+multipurpose sensor (9/15, 60%). For phase 2, with the addition of a smart speaker in the available devices to choose from, the majority of the participants chose the combination of door and window+multipurpose sensor+smart speaker (11/22, 50%).

**Table 3:** Internet-of-Things device selection by the participants.

Devices		Phase 1 (n=15), n (%)	Phase 2 (n=22), n (%)
Door and window sensor		12 (80)	15 (68)
Multipurpose sensor		14 (93)	18 (81)
IP^a^ camera		2 (13)	3 (14)
Smart speaker		N/A^b^	19 (86)
**Combinations of devices selected**			
	Door and window sensor only	1 (7)	1 (5)
	Multipurpose sensor only	3 (20)	0 (0)
	Smart speaker only	N/A	1 (5)
	Door and window+multipurpose sensor	9 (60)	2 (9)
	Door and window+multipurpose sensor+IP camera	2 (13)	0 (0)
	Door and window+multipurpose sensor+smart speaker	N/A	11 (50)
	multipurpose sensor+smart speaker	N/A	4 (18)
	IP camera+smart speaker	N/A	2 (9)
	Door and window+multipurpose sensor+IP camera+smart speaker	N/A	1 (5)

^a^IP: internet protocol.

^b^N/A: not applicable. Smart speaker was not offered during phase 1.

### Device Deployment and Maintenance

Over the course of the study, the primary investigator reviewed the status of the deployed sensor system remotely. The status of the sensor devices (door and window sensor and multipurpose sensor) was managed through the cloud remote management system of the Lab of Things platform. If the deployed system was offline, the primary investigator contacted the participants to schedule a maintenance visit. We recorded 22 maintenance visits outside the scheduled study visits throughout the study. In total, 11 maintenance visits were made to reboot the netbook used in the study. The netbook was used to receive and upload the sensor data for the deployment of a door and window sensor and multipurpose sensor and had to be left on all the time, 24/7, throughout the study. On some occasions, the netbook system froze due to memory overflow, and a manual reboot of the system was necessary. This issue was less of a problem for phase 2, where newer netbooks with bigger internal memory were used for the study. A total of 8 maintenance visits were made to re-establish the internet connection. One facility went through switching the internet service provider during the study; therefore, all the participants enrolled at that time from that specific building required an additional visit for setting up the devices.

### Feasibility of Data Collection

Overall, the study participants were able to easily complete the demographics and eHEALS questionnaires on their own during the baseline visit. Some participants mentioned that they were confused as several eHEALS items seemed repetitive. All health-related questionnaires (Instrumental Activities of Daily Living, Life-Space Assessment, and 12-item Short-Form Health Survey) were administered by the research team during the baseline and exit visits. There were no missing items in the questionnaire data collected. In one instance, a participant (ph2_p19) noted discomfort with the mental health–related questions in SF-12 but still provided responses. One participant (ph1_p7) declined to complete the exit questionnaires due to time constraints. Multimedia Appendix 2 shows the self-reported health-related parameters measured at the baseline and exit and the pre-post trends of these parameters. As expected, there were no statistically significant changes in any health-related variables between the 2-month study period.

### Participant Characteristics

Table 4 shows the demographic information of all study participants. There were no statistically significant differences in demographic parameters between the phase 1 and phase 2 participants. Overall, the participants in the study had a mean age of 78 (SD 9) years, were likely to be female (29/37, 78%) and have a Bachelor’s degree or higher (31/37, 86%). Four couples living together (n=8 married individuals) enrolled in the study together in phase 2 and the rest of the participants (n=29) in the study lived alone. The mean eHEALS score for participants was 32 out of a maximum of 40 (SD 6), indicating that the participants were generally comfortable using information technology for health situations. The majority of participants in the study had one or more self-reported chronic condition (33/37, 89%) and took more than 3 current medications (20/37, 54%). About half of the participants used some form of assistive devices (20/37,54%) such as a cane, a walker, or a wheelchair.

**Table 4:** Participant characteristics.

Characteristics		Phase 1 (n=15)	Phase 2 (n=22)	Combined (N=37)
Age (years), mean (SD)		77 (11)	78 (8)	78 (9)
Female, n (%)		12 (80)	17 (78)	29 (78)
**Marital status, n (%)**				
	Single	6 (40)	2 (9)	8 (22)
	Married or partnered	0 (0)	8 (36)	8 (22)
	Divorced	2 (13)	3 (14)	5 (14)
	Widowed	7 (47)	8 (36)	15 (41)
	Other	0 (0)	0 (0)	0 (0)
	Chose not to answer	0 (0)	1 (5)	1 (3)
**Education, n (%)**				
	Less than high school	0 (0)	0 (0)	0 (0)
	High school diploma or general education development	1 (7)	2 (10)	3 (9)
	Some college	1 (7)	1 (5)	2 (6)
	Bachelor’s degree	8 (53)	8 (38)	16 (44)
	Graduate or professional degree	8 (33)	10 (48)	15 (42)
eHEALS^a^ score, mean (SD; range)		35 (5; 26-40)	30 (7; 16-40)	32 (6; 16-40)
**Insurance, n (%)**				
	Medicare	15 (100)	22 (100)	37 (100)
	Medicaid	0 (0)	0 (0)	0 (0)
	Private insurance	6 (40)	9 (41)	15 (42)
	Other	2 (13)	9 (41)	5 (14)
**Number of chronic conditions (self-report), n (%)**				
	0	3 (20)	1 (5)	4 (11)
	1-3	7 (47)	18 (82)	25 (68)
	4+	5 (33)	3 (14)	8 (22)
**Number of current medications (self-report), n (%)**				
	None	4 (27)	2 (9)	6 (16)
	1-2	2 (13)	9 (41)	11 (30)
	3-4	5 (33)	4 (18)	9 (24)
	5+	4 (27)	7 (32)	11 (30)
**Use of assistive devices, n (%)**				
	Yes	10 (67)	10 (46)	20 (54)
	No	5 (33)	12 (55)	17 (46)

^a^eHEALS: electronic health literacy (8-40); higher scores represent higher self-perceived eHealth literacy.

### Summary of Interview Findings

In general, older adults showed a positive attitude toward IoT smart home technologies to support their health management. Many older adults commented that having such smart devices installed at their homes could help them better prepare for emergency situations. In addition, older adults showed an interest in having access to their activity level and environmental data collected by the sensors and discussed the benefits of using such data to monitor their health status and make informed decisions on their health management. Older adults who evaluated a smart speaker appreciated the convenience of a voice interface, as many used a smart speaker for setting up reminders and accessing the internet to retrieve information through the device. Along with the benefits, many noted their concern about privacy in using smart home technologies. The detailed findings of the qualitative assessment of the full interview data are beyond the scope of this paper and are presented in a separate paper (Choi et al 2020, unpublished document accepted at the *Journal of Gerontological Nursing*).

## Discussion

### Principal Findings

This pilot study evaluated the feasibility of using IoT smart home devices in real-world residential settings of older adults. The specific goal of this study is to assess some key aspects of trial design to inform future intervention studies using IoT smart home devices in older adults’ residences. Overall, our deployment results revealed that the use of IoT smart home devices is feasible in actual residences of older adults. Almost all participants (35/37, 95%) successfully completed the study protocol, and no major issues were identified during the study. In addition, the results show that older adults have varying degrees of acceptability to the different types of IoT smart home devices in real-world contexts. Most participants showed a preference for passive monitoring sensor devices and a smart speaker over IP cameras. Most participants considered an IP camera to be more intrusive and did not want it to be placed in their home environment. Our findings suggest that perceived privacy concerns, perceived usefulness, and curiosity to technology were strong factors when considering which device to have installed in their home. This aligns with some previous research that examined the acceptability of in-home sensor devices [14,18]. Future trials should consider older adults’ preferences for the different types and services offered by smart home devices to be installed in real-world residential settings [32].

### Recruitment and Retention

In this study, we collaborated with local retirement facilities in the Puget Sound area and recruited 37 people from 6 different retirement facilities. Our recruitment results show that the recruitment information session held at the retirement facilities was an effective strategy among the different recruitment activities in our study. One key benefit of the group information sessions was the reduced burden on the research team in the facilitation of the informed consent process. Future research should explore research partnerships with local retirement facilities and community agencies. The partnership could be mutually beneficial as research teams could gain easier access to potential older adult research participants, and the facilities could have increased access to innovative technology solutions and explore their potential applications in supporting their residents.

One barrier to participation was that some individuals who were interested in participating lacked internet access at home. Owing to limited funds and other practical constraints, we were not able to provide an internet connection and thus excluded those who did not have available access. This exclusion criterion could have turned away a group of participants who were not familiar with internet technology. No major challenges were noticed in our study procedures, and all but one participant failed to complete the study. The high retention could be explained by the low participant burden imposed by the study. In addition, engaging participants to choose the devices to evaluate at the start of the study may have eliminated any discomfort of having unwanted devices in their residence, in turn motivating them to remain in the study.

### Deployment Management

We identified some challenges to the maintenance of the deployed devices. We recorded a total of 22 additional visits to the participants’ homes outside the regular study visits. A total of 11 additional visits were necessary due to unforeseen technical issues, as we noticed that the memory overflow of the sensor data processing netbook required a manual reboot of the system. This issue was resolved through replacement of the netbook with ones with larger memory for the study. The issue of reliability and stability of the system deserves to be highlighted. The home gateway system that manages and controls interconnected IoT devices and the processing of the data received from the devices is an important central component of smart home infrastructure [33]. The reliability and stability of such home gateway systems for long-term operation is essential for designing future intervention studies that use IoT smart home technologies.

### Limitations

The primary study limitation is the generalizability of our findings due to a relatively small sample size with limited diversity in demographics recruited in a single metro area. Although the study team attempted to recruit from residential facilities across a breadth of socioeconomic status, the study sample was not able to cover a wide spectrum of older adults. The recruited sample did not include Medicaid beneficiaries and were highly educated compared with the general older adult populations. Therefore, the findings of the study must be interpreted with caution as the opinions on IoT smart home devices may vary in other regions or among broader demographics. In addition, the 2-month pilot deployment period may not be enough to understand the changes in perception and adoption behaviors over the long term. Furthermore, we only offer 4 different IoT monitoring devices for older adults to choose for this pilot study. The participants’ opinions might have varied had there been additional kinds of devices available for them. Despite these challenges, the data presented in this study can inform future studies exploring the use of smart home devices with older adults in their residential setting.

### Conclusions

Our study is particularly unique from previous studies, in that it assessed older adults’ preferences for different IoT sensor devices through real-world testing of IoT devices with older adults to address the literature gap. In addition, we combined environmental sensor data with motion sensor data to understand potential use cases of such integrated data in monitoring older adults’ activities. Furthermore, to our knowledge, our study is among the first attempts to explore the use of smart speakers in a health context with an older adult population. We believe the findings from this feasibility testing of an IoT smart home sensor system may identify barriers and limitations of the technology features critical to rapid adoption among older adults. This work will inform the follow-up assessment of IoT technologies and their impact on health-related outcomes and advance our understanding of the role of IoT home-based monitoring technologies to promote successful aging-in-place for older adults.

## Appendix

Multimedia Appendix 1 Overview of instruments and data collection schedule.

Multimedia Appendix 2 Health-related variables collected at baseline and exit.

## Abbreviations

eHEALS: eHealth Literacy Scale
IoT: Internet of Things
IP: internet protocol
