# Supplementary material for: Use of an Internet-of-Things Smart Home System for Healthy Aging in Older Adults in Residential Settings: Pilot Feasibility Study
Source: JMIR Aging. 2020 Nov 10;3(2):e21964. doi: 10.2196/21964 (PMC7685915; doi:10.2196/21964)
Supplement: Multimedia Appendix 1 [file aging_v3i2e21964_app1.docx]

Appendix 1. Overview of instruments and data collection schedule

| Types of Data Collected | | Instrument | Description | Schedule |
| --- | --- | --- | --- | --- |
| Participant characteristics | Demographics | Demographics questionnaire | at baseline age, gender, education level, marital status, insurance status, history of chronic conditions, current medications, and use of assistive devices | Baseline |
|  | Electronic health literacy | eHEALS^a^ | 8 item designed to measure consumers’ combined knowledge | Baseline |
| Health status indicators | Physical | SF-12v2^b^ | 6 item SF-12v2 PCS subscale | Baseline, Exit |
|  | Psychosocial | SF-12v2^b^ | 6 item SF-12v2 MCS subscale | Baseline, Exit |
|  | Activities of Daily Living | Lawton IADL^c^ | 8 domains of independent activities of daily living skills | Baseline, Exit |
|  | Mobility | Life Space Assessment^d^ | Extent of mobility and space occupied during the previous 4 weeks | Baseline, Exit |

a. eHEALS [23]

b. SF-12v2 [24], reliability to use among older adults [25]

c. Lawton IADL [26]

d. Life Space Assessment [27]
