# Supplementary material for: Use of an Internet-of-Things Smart Home System for Healthy Aging in Older Adults in Residential Settings: Pilot Feasibility Study
Source: JMIR Aging. 2020 Nov 10;3(2):e21964. doi: 10.2196/21964 (PMC7685915; doi:10.2196/21964)
Supplement: Multimedia Appendix 2 [file aging_v3i2e21964_app2.docx]

Appendix 2. Health-related variables at baseline and exit

|  | Phase 1  Baseline (n=15) | Phase 1  Exit (n=13) | Phase 2  Baseline (n=22) | Phase 2  Exit (n=22) | Combined sample at Baseline  (n=37) | Combined sample at Exit  (2-month)  (n=35) | *P*-value  (combined sample  baseline vs exit) |
| --- | --- | --- | --- | --- | --- | --- | --- |
| IADL^a^  (SD, Range) | 7.5  (0.9, 5.0-8.0) | 7.7  (0.9, 5.0-8.0) | 7.7  (0.8, 5.0-8.0) | 7.6  (1.1, 3.0-8.0) | 7.7 (0.7, 5-8) | 7.7 (1.0, 3-8) | 057 |
| LSA^b^  (SD, Range) | 52.9  (17.3, 27-82.5) | 55.3  (14.1, 36.0-76.0) | 64.2  (21.0, 28.5-100) | 60.0  (22.7, 16.5-100) | 60.5 (19.9, 28.5-100) | 58.2 (19.9, 16.5-100) | 032 |
| SF-12 PCS^c^  (SD, Range) | 42.7  (8.8, 21.6-58.5) | 43.3  (8.9, 25.7-56.8) | 46.7  (7.5, 32.0-56.5) | 52.9  (8.2, 31.4-56.1) | 45.08 (8.2 ,21.6-58.5) | 44.56 (8.4, 25.7-56.8) | 035 |
| SF-12 MCS^d^  (SD, Range) | 52.7  (8.7, 32.6-61.0) | 50.7  (7.0, 33.7-58.9) | 54.6  (6.6, 37.0-61.1) | 49.4  (9.1, 22.4-60.0) | 52.07 (7.4, 32.6-61.1) | 49.85 (8.3, 22.4-60.0) | 011 |

^a^IADL=Instrumental activities of daily living: 0 (low function, dependent) to 8 (high function, independent); LSA^b^=Life-Space Assessment of Mobility: A composite score ranges from 0 (mobility restricted to the bedroom) to 120 (independence enabling travel to out of town).; SF-12 PCS^c^ = 12-item Survey Physical Component Summary Measure, SF-12 MCS^d^ = Short form 12-item Survey Mental Component Summary Measure; Range 0 to 100, with higher score indicating better health
